# Physical activity practice among older adults: results of the ELSI-Brazil

**DOI:** 10.11606/S1518-8787.2018052000605

**Published:** 2018-10-25

**Authors:** Sérgio Viana Peixoto, Juliana Vaz de Melo Mambrini, Josélia Oliveira Araújo Firmo, Antônio Ignácio de Loyola, Paulo Roberto Borges de Souza, Fabíola Bof de Andrade, Maria Fernanda Lima-Costa

**Affiliations:** I Fundação Oswaldo Cruz . Instituto René Rachou . Núcleo de Estudos em Saúde Pública e Envelhecimento . Belo Horizonte , MG , Brasil; II Fundação Oswaldo Cruz . Instituto René Rachou . Programa de Pós-Graduação em Saúde Coletiva . Belo Horizonte , MG , Brasil; III Universidade Federal de Minas Gerais . Escola de Enfermagem . Departamento de Enfermagem Aplicada . Belo Horizonte , MG , Brasil; IV Fundação Oswaldo Cruz . Instituto de Comunicação e Informação Científica e Tecnológica em Saúde . Rio de Janeiro , RJ , Brasil

**Keywords:** Aged, Exercise, Sedentary Lifestyle, Epidemiologic Factors, Socioeconomic Factors, Idoso, Exercício, Estilo de Vida Sedentário, Fatores Epidemiológicos, Fatores Socioeconômicos

## Abstract

**OBJECTIVE:**

To describe the prevalence of the practice of physical activity (PA) among older Brazilian adults and associated factors. In addition, potential effect modifiers of the association between PA and age were investigated.

**METHODS:**

We have analyzed data from 8,736 participants (92.8%) aged 50 and older from the Brazilian Longitudinal Study of Aging (ELSI-Brazil). Physical activity was measured using the short version of the International Physical Activity Questionnaire. The outcome variable was defined as at least 150 minutes of weekly activities in all domains. The exploratory variables were age, sex, education, ethnicity, marital status, number of chronic diseases and medical appointments, and knowledge about or participation in public programs that encourage physical activity. Logistic regression and estimates of predicted probabilities were performed.

**RESULTS:**

The prevalence of recommended levels of physical activity was 67.0% (95%CI 64.3–69.5). Physical activity was associated with age [odds ratio (OR) = 0.97; 95%CI 0.96–0.98], higher educational level (OR = 1.27; 95%CI 1.11–1.45 for 4–7 years and OR = 1.52; 95%CI 1.28–1.81 for eight years or more), participants who were married/ in a long term relationship (OR = 1.22; 95%CI 1.08–1.38), and those who reported knowledge about (OR = 1.34; 95%CI 1.16–1.54) or participation in (OR = 1.78; 95%CI 1.34–2.36) a program aimed at the practice of physical activity. Women and those with lower educational level (p value for interaction < 0.05) reported lower physical activity levels.

**CONCLUSIONS:**

In addition to the association with marital status and health promotion programs, there were significant sex and educational level inequalities in physical activity decline later in life. These findings help the identification of groups more vulnerable to decreased physical activity levels with aging, as well as the planning of health promotion strategies, especially in older groups.

## RESUMO

**OBJETIVO:**

Descrever a prevalência da prática de atividade física entre adultos mais velhos brasileiros e os fatores associados a essa prática, além de identificar potenciais modificadores de efeito para a associação entre atividade física e idade.

**MÉTODOS:**

Foram analisados dados do Estudo Longitudinal da Saúde dos Idosos Brasileiros (ELSI-Brasil), compreendendo 8.736 indivíduos (92,8%) com 50 anos ou mais. A atividade física foi aferida pela versão reduzida do *Internati* *onal Physical Activi* *ty Questionnaire* . A variável desfecho foi a prática de pelo menos 150 minutos de atividades semanais em todos os domínios. As variáveis exploratórias foram idade, sexo, escolaridade, cor da pele, estado conjugal, número de doenças crônicas e consultas médicas, e conhecer ou participar de programas públicos de incentivo à atividade física. As análises foram baseadas na regressão logística e nas estimativas de probabilidades preditas.

**RESULTADOS:**

A prevalência de atividade física nos níveis recomendados foi de 67,0% (IC95% 64,3–69,5). A atividade física apresentou associação significativa com idade (OR = 0,97; IC95% 0,96–0,98) e foi mais comum na população com maior escolaridade (OR = 1,27; IC95% 1,11–1,45 para 4–7 anos e OR = 1,52; IC95% 1,28–1,81 entre aqueles com oito anos ou mais), entre os casados ou em união estável (OR = 1,22; IC95% 1,08–1,38) e entre aqueles que relataram conhecer (OR = 1,34; IC95% 1,16–1,54) ou participar (OR = 1,78; IC95% 1,34–2,36) de algum programa de incentivo à prática de atividade física. A redução da atividade física com a idade foi mais acentuada entre as mulheres e entre aqueles com menor escolaridade (valor de p para interação < 0,05).

**CONCLUSÕES:**

Além da associação com estado conjugal e programas de promoção da saúde, foi possível observar uma importante desigualdade em relação ao gênero e escolaridade na redução da atividade física com a idade. Esse conhecimento permite caracterizar grupos mais vulneráveis à redução da atividade física com o envelhecimento e contribui para o planejamento de ações de promoção da saúde, sobretudo nas faixas etárias mais velhas.

## INTRODUCTION

The global pandemic of physical inactivity has important social, economic, and health implications ^1^ . In a scenario of rapid population aging and increased burden of non-communicable chronic diseases (NCD), regular practice of physical activity (PA) is one of the most relevant strategies to improve health conditions in older groups, along with other health behaviors, such as having a healthy diet, smoking cessation, decreased excessive alcohol consumption, and overweight control ^1^ ^,^ ^2^ . Among older adults, the benefits of PA can be even more evident ^3^ , as it decreases the occurrence of NCDs, falls, and functional limitations and, consequently, leads to an increase in life expectancy and improved quality of life ^4^ ^-^ ^6^ .

However, worldwide levels of physical inactivity remain high ranging from 20% to 30% in the adult population ^7^ ^,^ ^8^ . In general, the proportion of individuals who do not reach the recommended minimum levels of PA is higher among older adults, women, and those in lower socioeconomic status ^7–10^ . In Brazil, despite a wide variation among studies, the prevalence of physical inactivity can be considered high among those aged 60 years and older and it ranges from 31% to 63% ^11^ ^,^ ^1^ ^2^ .

For the planning of programs that encourage the practice of PA, it is crucial to establish its determinants in different age groups to achieve well-being and healthy aging ^13^ . Evidence highlights the importance of social support, adequacy of physical activities to participants’ reality and interests, and knowledge about the risks and benefits associated to PA, considering the health status of older adults ^4^ ^,^ ^5^ . In the political context, the Brazilian Health Academy Program aims to overcome structural barriers to the practice of PA and adoption of healthy behaviors, especially among the most vulnerable groups ^14^ . This program is an important national strategy to encourage PA in the country ^15^ ^,^ ^16^ .

The identification of the determinants of PA and their relationship with aging could potentially help both the development and implementation of measures promoting this health behavior and, consequently, minimize the impact of NCDs later in life ^13^ . Although there is a consensus in the literature on the progressive PA decline with increasing age, the role of other variables as potential effect modifiers of this association remains poorly understood. The investigation of such effect modifiers will lead to identifying vulnerable groups and more specific initiatives.

The main objective of this study was to describe the prevalence of PA among older Brazilian adults and associated factors. In addition, potential effect modifiers of the association between PA and age were investigated.

## METHODS

### Data Source

The Brazilian Longitudinal Study of Aging (ELSI-Brazil) is a nationally representative, population-based cohort study of persons aged 50 years or more and their life and health conditions. The baseline survey was conducted between 2015 and 2016. For the random selection of the sample, municipalities were allocated to 4 strata depending on their population size. For the first 3 strata (municipalities up to 750,000 inhabitants), the sample was selected in 3 stages (city, census tract, and household). In the fourth stratum, which included the largest municipalities, the sample selection was done in 2 stages (census tract and household), including all cities. The planned number of interviews was 10,000 (9,412 participated), residing in 70 municipalities from different Brazilian regions. The data for this analysis were obtained through a face-to-face interview conducted at the participants’ household. More details can be found on the research homepage ^a^ and in another publication ^17^ .

The ELSI-Brazil was approved by the Research Ethics Committee of the *Fundação Oswa* *ldo Cruz* , Minas Gerais (CAAE 34649814.3.0000.5091), and all interviewees signed the informed consent forms to participate in the study.

### Study variables

The level of physical activity, the outcome variable, was measured using the short version of the IPAQ (International Physical Activity Questionnaire), translated and validated for Brazil ^18^ . This instrument contains questions related to the frequency (days per week) and duration (time per day) of the physical activities performed in the week before the interview, considering only those performed for at least 10 continuous minutes at a time, including: (a) walking (at home or at work; as transportation to go from one place to another; for leisure, for pleasure, or as exercise); (b) moderate activity (such as light cycling, swimming; dancing; light aerobics; amateur volleyball; lifting light weights; doing chores in the house, yard, or garden, such as sweeping, vacuuming, gardening, etc.; but, not including walking); and (c) vigorous activities (such as running; aerobics; soccer; fast cycling; basketball; lifting weights; doing heavy chores in the house, yard, or garden, etc.). We converted this information into total time of practice of PA in the reported week, considering double the time spent in vigorous activities. Regular PA level was defined as 150 minutes or more of PA per week, as recommended by the World Health Organization ^3^ .

The exploratory variables, selected according to literature ^9^ ^,^ ^14^ ^,^ ^15^ , included the following: age (in years), sex (male; female), educational level/schooling years (< 4; four to seven; eight or more), marital status (unmarried; married or common-law marriage), skin color (non-white; white), number of chronic diseases (zero; one; two or more), number of medical appointments in the past 12 months (zero; one or two; three or more), and participant’s knowledge about or participation in any public program that stimulates physical activity (no knowledge; knowledge but no participation; knowledge and participation). Their knowledge was evaluated by the following questions: “Do you know of any public program in your city that stimulates the practice of physical activity?” and “Do you participate in this program?”. Self-reported diagnosis of chronic diseases included: hypertension, diabetes mellitus, coronary heart disease (infarction, angina and heart failure), stroke, chronic lung disease, arthritis, depression, cancer and renal failure.

### Data Analysis

The descriptive baseline characteristics for the total sample and by their level of weekly physical activity used proportional distribution for categorical variables and mean and standard deviation (SD) for age, i.e., continuous variable. Comparison between the groups of PA was made using Student’s t-test or Pearson’s Chi-square test.

Logistic regression was used to estimate the odds ratio (OR) and respective confidence intervals (95%CI), without adjustment and mutually adjusted for all the explanatory variables. Using the logistic regression model, we also evaluated possible multiplicative interactions between each exploratory variable and age. The estimated predicted probabilities of practicing the recommended level of physical activity by age and variables with significant interaction, considering the adjustment for the other factors included in this analysis, were calculated. The results of the interactions were presented in charts.

All analyses were performed using the Stata package, version 14.0, using the procedures for complex samples, which include the sample weight of the individuals and the effect of the sampling design.

## RESULTS

This analysis included 8,736 individuals (92.8% of the total participants of the ELSI-Brazil) aged 50 years or more, who had information for all variables selected. The prevalence of regular physical activity based on recommended levels, i.e., at least 150 minutes per week, was 67.0% (95%CI 64.3–69.5).

Table 1 describes the characteristics of the studied population according to PA level. The sample had a mean age of 62.8 years (SD = 9.7 years); 53.5% were women, 36.9% had eight or more years of study, 63.9% were married or were in a common-law marriage, and 57.3% self-reported as non-white (black, brown, yellow, and indigenous). Two or more chronic diseases were reported by 39.8% of the participants and three or more medical appointments in the 12 months before the interview were reported by 48.9% of them. Most of the participants reported lack of knowledge about public programs that stimulate the practice of PA in the city of residence (56.8%) and only 5.7% knew and participated in these programs.

**Table 1:** Distribution of the characteristics of the studied sample, according to level of physical activity. Brazilian Longitudinal Study of Aging (ELSI-Brazil), 2015–2016.

Variable	Total ^a^	Level of weekly physical activity ^a^		p

		Recommended ^b^	Not recommended	
Age in years, mean (standard deviation)	62.8 (9.7)	61.6 (8.7)	65.1 (11.0)	< 0.001
Sex				
Female	53.5	52.8	54.8	0.237
Male	46.5	47.2	45.2	
Educational level in years				
< 4	31.4	27.3	39.8	< 0.001
4 to 7	31.7	32.0	30.9	
8 or more	36.9	40.7	29.3	
Marital status				
Unmarried	36.1	33.4	41.4	< 0.001
Married/Common-law marriage	63.9	66.6	58.6	
Race				
Non-white	57.3	56.3	59.3	0.138
White	42.7	43.7	40.7	
Number of chronic diseases				
Zero	27.1	28.6	24.1	< 0.001
One	33.1	33.5	32.2	
Two or more	39.8	37.9	43.7	
Number of medical appointments in the past 12 months				
Zero	16.7	16.9	16.5	0.205
1 to 2	34.4	35.2	32.8	
3 or more	48,9	47.9	50.7	
Knowledge and participation in a physical activity program				
No knowledge	56.8	53.2	64.2	< 0.001
With knowledge, but no participation	37.5	40.3	31.8	
Knowledge and participation	5.7	6.5	4.0	

^a^ Values expressed as percentage, unless otherwise specified.

^b^ Recommended level: at least 150 minutes/week, including walking and activities of moderate or vigorous intensity.

The recommended levels of PA was significant and reached by more young persons, those with a higher educational level, those married or in common-law marriage, those with fewer chronic diseases, and those who knew about or participated in programs of PA in the city ( Table 1 ).

Table 2 describes the magnitude of the association between the practice of PA at the recommended levels and the exploratory variables researched. After adjusting for all these variables, PA presented an inverse association with age, being less frequent among older adults, and it was more common in the population with a higher educational level, those married or in common-law marriage, and among those who reported knowing about or participating in some program that encourages the practice of PA.

**Table 2:** Association between practice of physical activity and sociodemographic variables, health conditions, use of health services, and participation in public physical activity programs. Brazilian Longitudinal Study of Aging (ELSI-Brazil), 2015–2016.

Variable	Recommended level of physical activity*	

	Crude OR (95%CI)	Adjusted OR (95%CI)
Age in years	0.96 (0.96–0.97)	0.97 (0.96–0.98)
Male (ref: female)	1.08 (0.95–1.24)	0.99 (0.85–1.16)
Educational level in years (ref: < 4)		
4 to 7	1.51 (1.30–1.76)	1.27 (1.11–1.45)
8 or more	2.03 (1.69–2.43)	1.52 (1.28–1.81)
Marital status (ref: unmarried)		
Married/Common-law marriage	1.40 (1.25–1.58)	1.22 (1.08–1.38)
Race (ref: non-white)		
White	1.13 (0.96–1.33)	1.07 (0.92–1.25)
Number of chronic diseases (ref: zero)		
One	0.88 (0.74–1.04)	0.96 (0.80–1.16)
Two or more	0.73 (0.62–0.87)	0.87 (0.71–1.06)
Number of medical appointments in the last 12 months (ref: zero)		
1 to 2	1.05 (0.89–1.24)	1.10 (0.92–1.31)
3 or more	0.92 (0.78–1.10)	0.98 (0.81–1.18)
Knowledge and participation in a physical activity program (ref: no knowledge)		
With knowledge, but no participation	1.52 (1.32–1.76)	1.34 (1.16–1.54)
Knowledge and participation	1.95 (1.47–2.60)	1.78 (1.34–2.36)

OR (95%CI): crude and adjusted odds ratio (95% confidence interval) for all variables listed in the table; Ref: reference

* Recommended level: at least 150 minutes/week, including walking and activities of moderate or vigorous intensity.

After adjusting for all variables, we found a significant interaction (p < 0.05) between age and sex, and between age and educational level. These results are shown in Figure . The practice of PA at the recommended levels decreases with increasing age, but this decrease was significantly more pronounced among women and among those with lower educational level (< 4 years). The other variables researched did not present significant interaction with age associated with the practice of PA.

**Figure:**
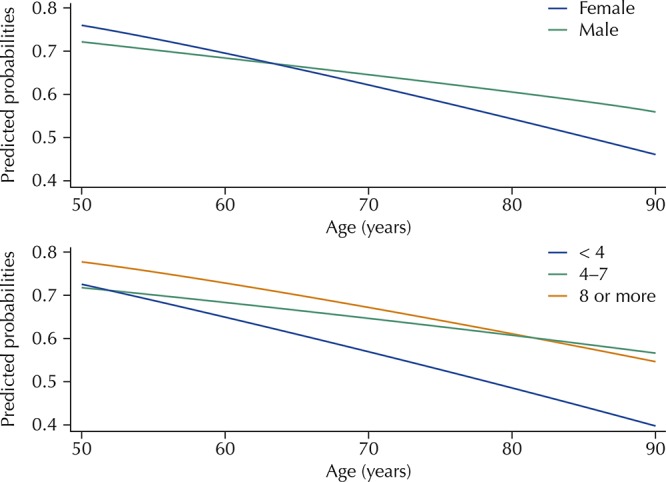
Predicted probabilities of practicing physical activity recommended by age, sex, and educational level. Brazilian Longitudinal Study of Aging (ELSI-Brazil), 2015–2016.

## DISCUSSION

Our main findings showed that the prevalence of older Brazilian adults achieving the recommended PA levels is 67.0%. Physical activity is inversely associated with age and is more frequent among those with high educational level, married, and those who know about or participate in public programs encouraging this practice. Declines in PA levels with increasing age were significantly more evident among women and in individuals with lower educational level.

The prevalence of PA among Brazilians varies widely and some of these differences can be attributed to the diversity of instruments used to measure this health behavior. Nevertheless, the results from ELSI-Brazil were similar to those found in older adults living in Bambuí, i.e., 68.8% were considered physically active ^11^ and in four areas of the state of São Paulo, where 73.9% reached moderate or high levels of PA ^19^ . At the national level, findings from the National Health Survey (PNS), conducted in 2013, showed that only 37.3% of the population aged 60 years or more reached the recommended levels of physical activity, which is lower than that observed in our study ^12^ . However, it is important to highlight that the PNS did not use the same instrument and did not include domestic activities, which may explain the differences observed in relation to our study that included activities carried out in all domains.

International studies have also shown wide variation in the estimates of PA prevalence. Baseline data from the Health and Retirement Study (HRS) investigating the American population aged 50 years or more, identified that 55.6% of the individuals engaged in vigorous activities or exercised at least three times per week ^20^ . On the other hand, the results collected in 16 European countries (Survey on Health, Ageing, and Retirement in Europe - SHARE) show an average prevalence of 87.5% of PA levels considered sufficient in the population aged 55 years or more, with wide variation between countries, with Portugal having the lowest prevalence (71.0%) ^21^ . Overall, the prevalence (67.0%) of PA at recommended levels in older adults found in our study is close to other Brazilian studies and within international variation.

The decline in PA levels with increasing age observed in older Brazilian adults is in accordance with that reported in the literature ^9^ ^,^ ^12^ ^,^ ^13^ ^,^ ^21^ , despite its recognized benefit for healthy aging ^3^ ^,^ ^4^ ^,^ ^6^ ^,^ ^22^ . Lower PA levels among older women and in the worst socioeconomic groups, similar to our findings, have also been reported in other studies ^9^ ^,^ ^13^ ^,^ ^19^ ^,^ ^23^ . However, the results of the ELSI-Brazil show an interaction between these variables and age, which allows the identification of more vulnerable groups and, therefore, the planning of interventions more tailored to these groups. We found that the practice of PA at the recommended levels decreased with increasing age, but this decline was significantly more pronounced among women and those with the lowest educational level (< 4 years).

Overall, older women report more barriers to PA compared to men, including lack of companion, lack of interest and health problems ^24^ ^,^ ^25^ , which may reflect both the greater survival of this group and explain the greater PA decline among women with increasing age. In addition, evidence from English women aged 60 to 79 years has shown that socioeconomic status at different stages of the life course determined PA in this age group ^26^ . Although our results are adjusted for educational level, it is plausible that other aspects of the socioeconomic condition could act differently in women leading to a greater perception of barriers that would hinder the practice of PA, such as worse evaluation of the environment, lack of safety, poorer health and lack of social support ^24^ ^,^ ^25^ .

It is important to mention that possible mechanisms explaining greater prevalence of health risk behaviors in lower socioeconomic level groups may change over time. The lack of knowledge or resources in this group to modify harmful health behaviors may be less important because of the wide dissemination of the role of these risk factors on health conditions in the mass media and greater access to education, health services, and public programs that encourage a healthy lifestyle ^27^ . However, differences in social support and motivation to effectively produce changes in health behaviors may still persist and explain the greater vulnerability of lower education groups towards PA ^27^ . Data from the Health and Retirement Study have shown that, although common in persons aged 50 years or more and recently diagnosed with a health condition, behavioral changes, such as stopping smoking and starting PA, are more common in higher educated individuals ^23^ . This may increase the differences in PA with increasing age, as observed among older adults from ELSI-Brazil. A prospective study conducted in the Netherlands has shown that low educational level was a determining factor in leisure-related PA declines. This association was explained in individuals with lower educational level because of material problems and the worse perception of control and health ^28^ ; these factors may also clarify the association and also be the target of health promotion programs in this specific group.

We found that married or individuals in common-law marriage were more likely to reach the recommended PA levels, which indicates a possible role of social support for this practice, especially among older adults ^4^ ^,^ ^25^ . Studies on the reported barriers to start or maintain adequate levels of PA have shown the importance of the participation of friends, family members, or any other company for PA ^21^ ^,^ ^25^ , which suggests that our results may represent this association.

We found a higher prevalence of recommended levels of PA among individuals who reported knowing about or participating in any public program that encourages this practice, even after adjusting for other factors. In Brazil, the Health Academy Program is a key strategy to control NCDs adopted by the Ministry of Health, and other studies have already demonstrated the importance of the knowledge about or participation in this program to achieve the recommended levels of leisure-related PA ^15^ ^,^ ^16^ . In Belo Horizonte, Brazil, leisure-related PA was more frequent among adults living near the centers of this program, which highlights the potential of this strategy in influencing the practice of PA, even among non-users ^29^ .

Some studies have shown lower frequency of PA among individuals reporting NCDs ^19^ ^,^ ^22^ , besides the importance of medical advice as a facilitator for the regular practice of PA ^25^ . However, the results of our study did not show an association between number of chronic diseases, number of medical appointments, and recommended levels of PA. This may suggest greater importance of other factors, such as age and educational level in terms of PA. Nevertheless, it is important to recognize the impact of PA on the prevention of NCDs, functional limitations and mortality among older adults ^3–5^ , which makes this practice a therapeutic alternative for individuals diagnosed with chronic diseases ^6^ . A study conducted in 41 cities in the Southern and Northeastern regions of Brazil has shown that this advice was less frequent in the population aged 80 years or more and among sedentary individuals ^30^ , which indicates the greater vulnerability of these groups in relation to the lack of incentive by health services and professionals.

Among the limitations of this study, we need to consider the sectional design, which does not allow establishing temporal relations between the investigated variables, and the use of self-reported information. Regarding the instrument used to measure PA in this study, the IPAQ does not differentiate domains and we could not evaluate whether the associations occur differently in each PA domain, as previously observed in the Brazilian adult population ^31^ . However, we highlight that we analyzed a large representative sample of older Brazilian adults using standardized procedures and a validated instrument (IPAQ) widely used to measure PA, which demonstrates the profile of this behavior in the country for older adults.

In summary, the results presented allow the identification of the most vulnerable groups that should be targeted by specific interventions, which may reduce the global burden of chronic diseases in a scenario of fast population aging. Women and those with the lowest educational level had considerably greater PA decline with increasing age. In addition to individual factors, PA is inserted in a broader context, involving environmental determinants and, therefore, it should also be the target of health promotion policies, such as public programs that encourage the adoption of healthy habits.

## Footnotes

a Fundação Oswaldo Cruz. Brazilian Longitudinal Study of Aging. Rio de Janeiro; c2015 [cited 2017 Nov 28]. Available from: http://elsi.cpqrr.fiocruz.br
